# *N*-acetyl-*L*-leucine in Neurological Disorders: Mechanisms, Evidence, and Therapeutic Perspectives

**DOI:** 10.1007/s12311-026-01996-2

**Published:** 2026-04-28

**Authors:** Mobeen Abid, Shanzay Akhtar, Hammad Amjad, Sabeeh Khawar Farooqui, Tasbiha Naz, Saad Shakil, Hamzah M. Alghzawi, Areesha Hafeez, Anja Vossenkaul, Chiara Brechbuehl, Tatiana Bremova-Ertl

**Affiliations:** 1 Karachi Medical and Dental College, Karachi, Pakistan; 2 Ziauddin University, Karachi, Pakistan; 3 Department of Medicine, Ziauddin University, Karachi, Pakistan; 4 School of Nursing, Tennessee State University, Nashville, TN USA; 5 Department of Pediatrics, University Hospital Inselspital, Bern, Switzerland; 6 Department of Neurology, Department of Neuropediatrics and Center for Rare Diseases, University Hospital Inselspital, Rosenbühlgasse 25, Bern, 3010 Switzerland

**Keywords:** *N*-acetyl-*L*-leucine, Niemann-pick disease type C, GM2*-*Gangliosidoses, Ataxia Telangiectasia, Multiple sclerosis, Parkinson’s disease

## Abstract

*N*-acetyl-*L*-leucine (NALL), a stereospecific derivative of the essential amino acid L-leucine, has attracted increasing attention as a candidate therapy for neurological disorders. Preclinical and clinical studies suggest both symptomatic and neuroprotective effects. This review summarizes evidence on the mechanisms of action, preclinical findings, and clinical outcomes of NALL, and discusses its therapeutic prospects in neurological health and disease. A structured literature search of PubMed, Scopus, and Google Scholar was conducted to identify preclinical and clinical studies evaluating N-acetyl-L-leucine (NALL) in neurological disorders. Preclinical studies demonstrated that NALL enhances mitochondrial bioenergetics, reduces oxidative stress, restores lysosomal and autophagic function, and modulates neuroinflammation. In rodent models, NALL improved motor coordination, neuronal survival, and recovery following traumatic brain injury. Clinical trials in Niemann-Pick disease type C and GM2 gangliosidoses showed improvements in motor function, quality of life, and disease progression, with sustained benefit during extension phases. Preliminary evidence also suggests possible roles in multiple sclerosis, ataxia telangiectasia, Parkinson’s disease, and multiple sulfatase deficiency. NALL was consistently well tolerated with a favorable safety profile. NALL shows multimodal mechanisms and promising translational potential for both rare and common neurological disorders. While early evidence is encouraging, larger randomized trials are required to confirm efficacy, define optimal dosing, and determine its place in neurological therapeutics.

## Introduction

Neurodegenerative disorders are one of the most threatening to global health in the current century, encompassing a range of disorders that directly affect the nervous system, reducing its structure and functionality. Conditions such as Parkinson’s disease (PD), multiple sclerosis (MS), Alzheimer’s disease and other dementias, and motor neuron disease exemplify this growing burden [1].These chronic disorders such as MS and PD have affected more than 2.3 million and 6 million people around the world, respectively [2–4]. Not only are common neurological diseases (CNDs) on the rise, but also rare (“orphan”) neurological diseases (RNDs). For the past thirty years, there has been an increase of 39% in the number of deaths related to neurodegenerative and neurological disorders with a simultaneous increase of 15% in disability-adjusted life-years despite the measures to control the spread of diseases [2].

Beyond these more prevalent disorders there is a diversity of rare neurodegenerative diseases, in which ensuring that research continues, enhancing diagnostic precision are important but often under-resourced endeavours [5]. As neurodegenerative disorders become an increasingly large public health issue, new therapies are being introduced to the market to treat both the common and rare disorders. N-acetyl-L-Leucine (NALL) represents one of the most promising candidates. This review highlights the role of NALL in neurological health, discussing key molecular pathways, preclinical and clinical evidence, and emerging therapeutic prospects.

## Literature Search Strategy

### Review Question

The research question of the current study was *“what evidence is present in published literature regarding N-acetyl-L-leucine and its symptomatic and neuroprotective benefit in neurological disorders and how consistent and reproducible is the evidence from a preclinical to a clinical setting?”.* The aim of the current review was to, (1) map and summarize molecular mechanisms, preclinical findings and clinical trial data on the role of NALL on neurological health and disease and (2) provide perspectives on its therapeutic potential, safety profile and future implications for managing neuroinflammatory and neurodegenerative conditions.

### Search Strategy and Study Selection

A structured literature search was conducted by M.A. and S.A., by screening scientific online databases (PubMed, Scopus, and Google scholar) to identify relevant studies, using the following search string: *(“N-acetyl-L-leucine” OR “N-acetylleucine” OR “acetyl-L-leucine” OR “NALL” OR “IB1001”)*. Studies reporting mechanistic, preclinical, or clinical data on NALL in neurodegenerative and related neurological disorders were considered. These included lysosomal storage disorders (such as Niemann–Pick disease type C and GM2 gangliosidosis), hereditary ataxias including ataxia-telangiectasia, Parkinsonian syndromes, multiple sclerosis, and multiple sulfatase deficiency. Studies were selected based on their relevance to the objectives of this narrative synthesis and their contribution to understanding the neuroprotective and therapeutic potential of NALL. The studies included were from the time period of January 2000 to October 2025. No other restrictions on document type or language of records were posed.

This review was not conducted as a formal systematic review or meta-analysis; therefore, no quantitative synthesis or structured risk-of-bias assessment was performed, given the heterogeneity of study design and outcomes.

### Evidence Screening and Charting

Identified records were screened for relevance based on title and abstract. Full-text articles were reviewed when necessary to confirm their applicability to the objectives of this narrative review. Data from selected studies were summarized descriptively, focusing on study design, patient population, intervention characteristics, and reported outcomes. Key clinical studies are presented in Table 1 to provide an overview of available human evidence on N-acetyl-L-leucine in neurodegenerative disorders.

**Table 1:** Summary of clinical trials of *N*-acetyl-*L*-leucine

Study (Year) [Ref]	Disease(s)	Phase/Study Design	Administration	Population	Intervention (Route, Dosage)	Duration/Follow up
Bremova-Ertl, T, 2024 [18]	NPC	Phase III, randomized, double-blind, placebo-controlled	Oral	Patients ≥ 13 y	Oral, 2–4 g/day (≥ 13 y: 4 g/day)	12 weeks
Bremova-Ertl, T, 2021 [21]	NPC	Phase IIb, randomized, double-blind	Oral	Patients ≥ 13 y; children 6–12 y (weight-tiered)	Oral, 4 g/day (weight-based for 6–12 y)	12 weeks
Patterson MC 2025 [22]	NPC	Multinational study: randomized, double‑blind, placebo‑controlled crossover trial	Oral	53 pediatric and adult patients (age 5‑67 y; ≈45% female)	N‑acetyl‑L‑leucine granules orally; patients ≥ 13 y or children 4–12 y weighing ≥ 35 kg received 4 g/day; children < 35 kg received weight‑tiered doses approximating 0.1 g/kg/day	Open‑label extension lasted ≥ 1 year with visits at 6 and 12 months
T Fields, 2021 [19]	NPC, GM2, or A-T	Phase II, open-label, multi-center	Oral	Patients ≥ 13 y; children 4–12 y (weight-tiered: 2–4 g/day)	Oral, 2–4 g/day (age- and weight-adjusted)	12 weeks
Mehran Beyraghi-Tousi 2024 [23]	AT	Pilot study, open-label, underpowered case series	Oral	Small cohort (*n* < 10)	Oral, 4 g/day	6 weeks
Kyriakos Martakis 2023 [51]	GM2	Phase II, open-label	Oral	Patients ≥ 13 y; children 6–12 y (weight-tiered)	Oral, 4 g/day	6 weeks
. Kremmyda O 2023 [80]	MS	Case report	Oral	Single patient	Memantine 20 mg/day (ongoing) plus acetyl‑DL‑leucine 3 g/day for three weeks, then increased to 5 g/day	Combination therapy given for three weeks; long term (stable at five years)
Xu Z 2023 [20]	Parkinson’s disease	Animal Trial	Oral	Male C57BL/6 J mice, aged six weeks and weighing 18 ± 2 g	N‑acetyl‑L‑leucine (NALL) administered orally	-
Saberi-Karimian et al., 2022 (IRCT20210413050958N5) [24]	MSD	Proof-of-concept, N-of-1 crossover	Oral	Single patient	Oral, 3 g/day (1 g TID)	4 weeks per treatment stage (crossover) — N-of-1 proof-of-concept

*NPC* Niemann–Pick disease type C, *GM2* GM2 gangliosidosis, *A-T* ataxia-telangiectasia, *MS* multiple sclerosis, *PD* Parkinson’s disease, *MSD* multiple sulfatase deficiency, *CBC* complete blood count, *TID* three times daily, *UK* United Kingdom, *USA* United States of America

## Results

The structured literature search identified a limited number of preclinical and clinical studies directly evaluating NALL in neurological disorders. The available evidence includes animal models, small open-label trials, case reports, case series and phase II-III randomized studies primarily in lysosomal storage disorders. Given the heterogeneity of study designs and outcomes, findings are presented descriptively.

## Mechanisms of Action of *N*-acetyl-*L*-leucine

**Fig. 1:**
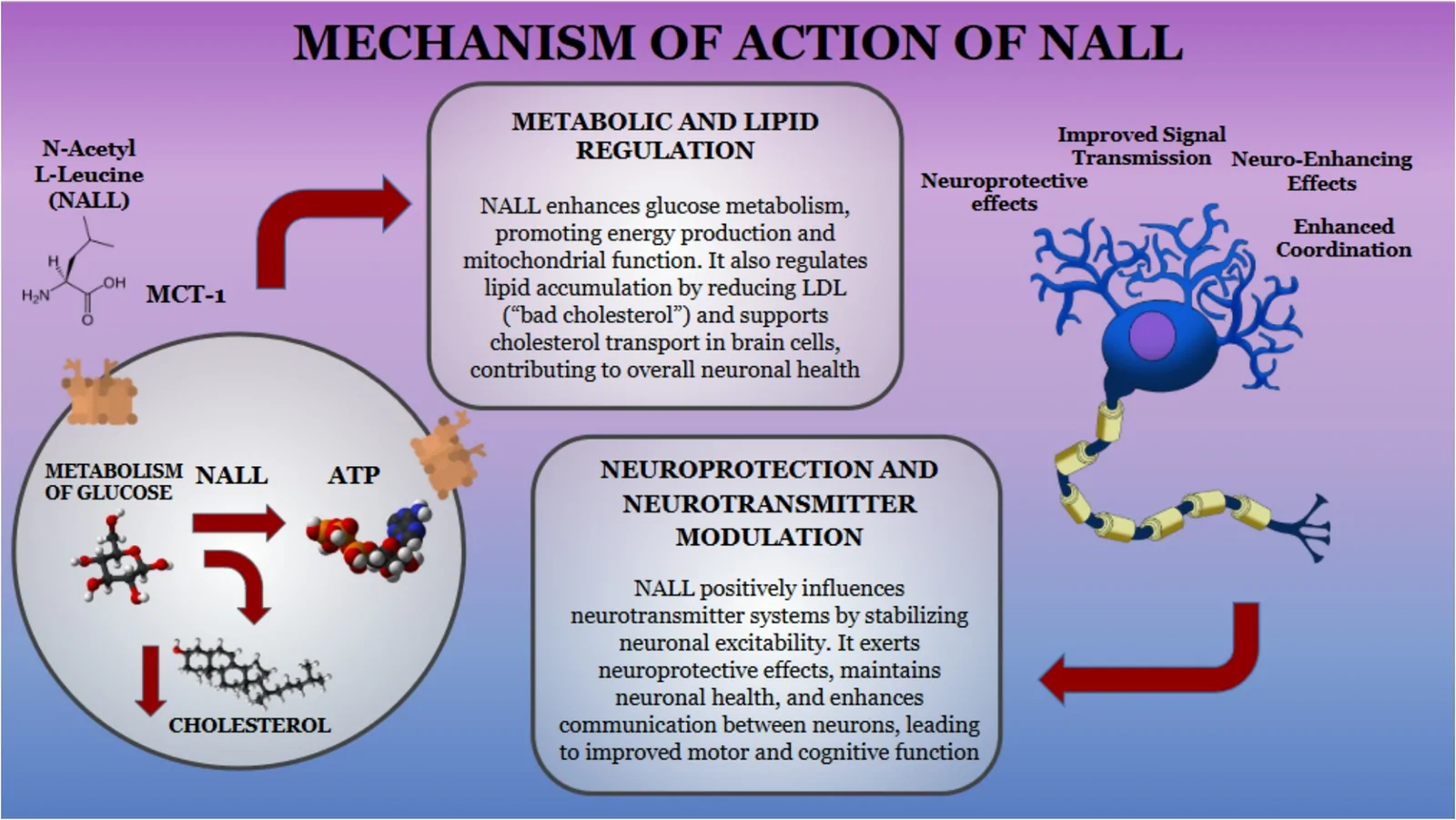
Mechanism of action of *N*-Acetyl-* L*- leucine

NALL is the L-enantiomer of the racemate *N*-acetyl-*DL*-Leucine. *N*-acetyl-*DL*-Leucine is an acetylated derivative of the amino acid leucine, which has been used in France since 1957 as a treatment for vertigo. Unlike this racemic mixture previously used for vertiginous symptoms, the L-enantiomer (NALL) has demonstrated therapeutic benefits in neurological conditions such as lysosomal storage disorders, particularly Niemann-Pick disease type C (NPC) [6]. The neuroprotective and symptomatic effects of NALL are attributed to multiple interconnected biological pathways. Figure 1 illustrates the proposed cellular pathways influenced by NALL.

### Transport Mechanism

The acetylation of NALL is intrinsic to its pharmacological activity. The N-acetyl group of NALL renders it a substrate for membrane-spanning monocarboxylate transporters (MCTs), delivering supraphysiological levels of NALL relative to leucine into the cytoplasm. As MCTs are ubiquitously expressed, NALL is taken up and delivered to all tissues. Once inside cells, it is deacetylated yielding L-leucine, which affects cell metabolism by entering enzyme-controlled pathways that correct metabolic dysfunction and improve energy production without deleterious effects [7, 8]. 

### Mitochondrial Function and Energy Metabolism

Neurological disorders, especially neurodegenerative diseases, are often characterized by mitochondrial dysfunction leading to impaired energy production. NALL has been shown to enhance mitochondrial bioenergetics by promoting mitochondrial respiration and biogenesis, thereby restoring adenosine triphosphate (ATP) levels essential for neuronal function and survival [9]. Recent trials highlight NALL’s ability to shift glucose metabolism to yield more ATP, as shown in NPC mouse models.

Notably, NALL has been shown to correct mitochondrial dysfunction/energy metabolism irrespective of whether there is insufficient or excessive Krebs cycle flux (anaerobic or aerobic respiration) [10]. While disorders like NPC are characterized by a shift towards using glycolysis and therefore less flux through the Krebs cycle, in diseases like GM2 gangliosidosis, neurons cannot make use of anaerobic pathways and therefore have abnormally high use of the Krebs cycle, a byproduct of which is oxidative stress. The buildup of reactive oxygen species (ROS) harms cellular components in neurons. NALL demonstrates antioxidant capabilities by neutralizing free radicals and boosting natural antioxidant defenses such as glutathione, thus reducing oxidative harm to neurons. Comparable antioxidant pathways have been observed for other N-acylated substances such as N-acetyl serotonin (NAS), which demonstrated the ability to lower ROS levels, elevate glutathione, and promote antioxidant enzymes through *Nrf2* signaling in neuronal models under glutamate stress [11]. Despite their structural differences, the common N-acyl modification in NAS and NALL may contribute to similar antioxidant properties important for neuroprotection in oxidative stress scenarios.

### Lysosomal Function and Substrate Accumulation

Neurological disorders, especially neurodegenerative diseases, are often characterized by lysosomal dysfunction leading to substrate accumulation. NALL rapidly and stereospecifically activates Transcription Factor EB (TFEB) which is the master regulator of lysosomal biogenesis and autophagy, driving *TFEB* nuclear translocation and induction of *CLEAR* network genes. Functionally, this increases lysosomal mass and competency and enhances autophagic flux, changes that are expected to promote clearance of stored substrates and restoration of lysosomal degradative capacity [6, 8].

NALL also acts at the level of inter-organelle communication. In NPC patient cells with coupled mitochondrial–lysosomal dysfunction, NALL reverses lysosomal pathology and mitochondrial defects in parallel, consistent with improved mitochondria–lysosome crosstalk at contact sites and downstream correction of organelle stress signaling. This inter-organelle rescue likely cooperates with *TFEB* activation to re-establish lysosomal acidity/turnover and reduce substrate buildup [10] Preclinical data in NPC and GM2 mouse models provided by Kaya et al. also align with these mechanisms and demonstrated that Acetyl L-Leucine (and the racemate) delays disease progression and synergizes with substrate-reduction therapy (miglustat), consistent with reduced substrate burden and improved cellular bioenergetics when lysosomal function is restored [6].

Together, these actions of NALL reduce pathological lipid accumulation and restore lysosomal homeostasis in NPC and related lysosomal storage disorders [6, 8, 10].

### Neuroinflammation Modulation

Chronic neuroinflammation exacerbates neuronal damage in conditions such as MS, Alzheimer’s disease, cerebellar and neurodegenerative ataxias. NALL demonstrates anti-inflammatory effects by downregulating pro-inflammatory cytokines and reducing microglial activation [12]. These neuroprotective effects have been confirmed in several trials reporting a significant reduction in neuroinflammation following NALL treatment [6, 12, 13].

### Ion Channel and Membrane Stabilization

Precise ion channel function and membrane potential regulation are critical for neuronal excitability. Amino acids like NALL influence ion channels and stabilize the membrane potential, potentially reducing excitotoxicity, a mechanism implicated in stroke and traumatic brain injury. A study by Torregrosa et al. reinforces this [14]. On the cellular level, improved ATP production via NALL treatment helps prevent depolarization-related impairments in membrane excitability, thereby preserving neuronal communication [15].

### Modulation of Neurotransmitter Systems

Preliminary evidence suggests that NALL may impact neurotransmitter systems, particularly glutamate, thereby improving neuronal signaling and potentially reducing excitotoxicity [16].

### Emerging Role in Autophagy

Multiple studies have identified NALL restores autophagic flux, including models of NPC and Traumatic Brain Injury, contributing to NALL’s neuroprotective actions [12, 17].

## Preclinical Evidence (Animal Models and Cellular Studies)

Preclinical studies provide compelling data on the symptomatic and neuroprotective effects of NALL:

### Ataxia and Cerebellar Degeneration

In models of hereditary cerebellar ataxias such as NPC, NALL improved motor coordination, balance, and neuronal survival, including when mice were treated acutely (e.g. symptomatic treatment effect) and pre-symptomatically (a neuroprotective, disease-modifying effect). In *Npc1⁻/⁻* mouse models, treatment with NALL delayed the onset of functional decline (gait abnormalities, motor dysfunction), slowed disease progression, and prolonged survival [6]. These beneficial effects were specific to the L-enantiomer and were not observed with N-acetyl-D-leucine. The effective dose (0.1 g/kg/day) in mice corresponds to the dose used in clinical trials [18]. 

### Traumatic Brain Injury

In rodent models of traumatic brain injury (TBI), post-injury oral administration of NALL markedly enhanced cognitive and motor results, decreased lesion volume, and diminished neuroinflammatory responses. These effects were linked to reduced neuronal cell death and a partial recovery of autophagy flux, indicating the involvement of NALL in enhancing neuroplasticity and neuroprotection after injury [12].

### Neuroinflammation and Neurodegeneration

Preclinical research consistently indicates that N-acetyl-L-leucine exerts neuroprotective benefits through several biological mechanisms relevant to both neuroinflammation and neurodegeneration. Cellular and animal studies indicate that NALL provides neuroprotection by enhancing mitochondrial, lysosomal, and synaptic proteins in dopaminergic neurons, while also decreasing neuroinflammation through the partial recovery of autophagy following injury [9, 12]. Marked decrease in cortical neuronal death and attenuated inflammatory signaling was reported in experimental mouse models with traumatic brain injury being administered NALL, an effect linked to restoration of impaired autophagy flux, a critical pathway for cellular homeostasis and removal of damaged proteins and organelles [12].

These observations imply that NALL targets upstream drivers of neurodegeneration. Additional experimental data indicate that NALL modulates microglial activation within cerebellar tissue, an important observation because activated microglia contribute directly to neuronal loss through inflammatory cytokine release and phagocytic damage to synapses and axons [19]. In neurodegenerative disease models, particularly Parkinsonian mice exposed to MPTP toxin, NALL demonstrated neuroprotection associated with modulation of the gut–brain axis and suppression of inflammatory microbiome taxa such as desulfobacterota, suggesting systemic immune regulation as an additional mechanism of action [20]. NALL reduced microglial activation and pro-inflammatory cytokine levels, suggesting its role in modulating neuroinflammatory pathways pertinent to diseases including MS and Alzheimer’s [12] Cellular and animal studies indicate that NALL provides neuroprotection by enhancing mitochondrial, lysosomal, and synaptic proteins in dopaminergic neurons, while also decreasing neuroinflammation through the partial recovery of autophagy following injury [9, 12].

## Clinical Evidence (Emerging Human Studies)

Although large-scale randomized controlled trials remain limited, several promising clinical findings have been reported:

### Hereditary Cerebellar Ataxia and Niemann-pick disease type C

In a phase 2b trial that was assessor-blinded and multinational, NALL notably enhanced the functioning and quality of life (QoL) in adults and children with NPC following 6 weeks of treatment. Improvements included decreased ataxia, improved walking and gait coordination, improved fine motor skills, and stabilization of neurological manifestation such as dysarthria and cognitive deterioration [21]. A subsequent phase 3 randomized, placebo-controlled study validated these results during a 12-week treatment period, demonstrating sustained enhancements versus placebo in neurological manifestations, functioning, and QoL and a positive safety profile [18]. The findings from the extension phase after 18-months also correlate to a significant disease-modifying, neuroprotective effect of NALL [22].

### Traumatic Brain Injury

There have been no published human clinical or observational studies on NALL in TBI to date. The present results are confined to rodent models, where oral NALL enhanced motor and cognitive results and decreased neuroinflammation post controlled cortical impact injury [12].

### Neuroinflammation and Neurodegeneration

Clinical data suggests translational relevance of the mechanistic effects including neuroinflammation and neurodegeneration. A randomized control trial involving ataxia telangiectasia reported that NALL did not significantly improve primary motor endpoint, although it did improve secondary systemic symptoms such as nausea and constipation, suggesting that neurological benefit may be influenced by factors such as disease stage, duration of therapy, or patient selection [23]. Preliminary clinical observations suggest that NALL exerts anti-inflammatory and neuroprotective effects in patients with neurodegenerative diseases. A decrease in pro-inflammatory markers such as interleukin-6 (IL-6) has been observed in initial human studies, suggesting possible immunomodulatory advantages awaiting further confirmation via controlled trials [24, 25]. Expert commentary in a New England Journal of Medicine publication further identifies NALL as a promising investigational candidate for neurodegenerative conditions, citing its strong mechanistic rationale and encouraging early findings while emphasizing that larger, well-powered trials are necessary to establish definitive disease-modifying effects [13]. A clinical crossover case study in patients with multiple sulfatase deficiency reported neurological improvement and cited prior evidence that N-acetyl-L-leucine reduces cerebellar inflammation, supporting the hypothesis that anti-inflammatory activity observed in animal models may also occur in humans [24].

## Neurological Disorders

### Niemann-pick Disease Type C

#### Epidemiology and Etiology

NPC, an autosomal recessive, neurovisceral lysosomal storage disorder that manifests as neurological, systemic, and psychiatric symptoms [18]. This highly heterogeneous, pan-ethnic, ultra-rare, prematurely fatal disease has an occurrence ranging from perinatal to late adulthood, with most patients eventually developing fatal neurological disease [26]. Approximately 8% of patients who are diagnosed as progressive supranuclear palsy (PSP) are found to have NPC1, highlighting potential for diagnostic overlaps and importance of genetic or biochemical confirmation [27].

#### Pathophysiology

NPC is characterized by mutations in the *NPC1* and/or *NPC2* genes leading to endolysosomal storage of unesterified cholesterol in the liver, brain, and other tissues [26]. Certain regions of the brain, such as the Purkinje cells of the cerebellum, are affected more severely. In these neurons, there is storage of free sphingosine, glycolipids and gangliosides, being the dominant lipids accumulated. The stored cholesterol causes secondary alterations of sphingomyelin metabolism in the neural and extra neural tissues, resulting in neuroinflammation, neurofibrillary tangles, neuroaxonal dystrophy and cell death [7, 28]. NPC combines lipid toxicity with disrupted cholesterol homeostasis, leading to both cellular dysfunction and systemic neurological decline [28].

#### Clinical Manifestations

Typically, NPC is a subacute form with central nervous system (CNS) involvement and moderate sphingomyelin accumulation [29]. Disease severity is defined by neurologic involvement and preceded by systemic signs, including cholestatic jaundice and hepatosplenomegaly [26]. Key features include cognitive decline, ataxia, cataplexy, vertical gaze palsy, seizures, psychiatric symptoms, and dystonia [26, 30]. Although the presentation varies, the disease is always progressive, leading to premature death or severe disability without effective management [28].

#### Role of N-acetyl-L-Leucine in Niemann-pick Disease Type C

NALL demonstrates neuroprotective and disease-modifying effects in treating NPC [21]. NPC disrupts ATP generation, which is an essential component of cellular function. NALL enters enzymatic pathways and improves overall cellular energy by restoring ATP levels [18]. In addition, ATP depletion also affects neuronal communication, causing issues with membrane excitability and ion transport. Studies have demonstrated NALL to improve neuronal membrane potential, restoring proper signaling and safeguarding neuronal circuits improving motor function and slowing down disease progression [6, 15, 31].

Further investigations in children and adults in various clinical trials also support the significant positive outcomes by treatment with NALL leading to improved QoL, symptom reduction and better overall functioning [18, 21, 32]. To determine the potential dampening of neurodegenerative effects, clinical and pre-clinical trials observed their assessment through various protocols including modified disability scale (mDRS), Scale for the Assessment and Rating of Ataxia (SARA) and Spinocerebellar Ataxia Functional Index (SCAFI) [18, 21, 32]. SARA and SCAFI are two modes administered to assess the cerebellar functions, SARA is an eight-item clinical rating scale comprising of gait, stance, sitting, speech, finger-chase test, finger-nose test and fast alternating hand movements, whereas SCAFI includes 8-meter walking test (8MWT) and 9-Hole Peg Test (9HPT). The mDRS is a six-domain system including manipulation, seizures, ambulation, swallowing, language and ocular movements, used to evaluate neurologic status [32]. To evaluate the efficacy and safety of NALL, two clinical trials, NCT03759639 and IB1001-301, were conducted, and the SARA, mDRS, and SCAFI scores were assessed among the participants. SARA is a validated clinical tool for the assessment of spinocerebellar ataxias, and the scores of improvements were consistent in both studies. The mDRS and SCAFI scores statistically improved in patients after the trial [18, 21].

#### Strengths and Weaknesses of N-acetyl-L-Leucine in Modifying Disease Course of Niemann-pick Disease Type C

NALL shows promise as a disease modifying therapy for NPC. A recent placebo-controlled randomized control trial demonstrated improved neurologic function after 12 weeks, reflected by marked reduction in ataxia severity score (− 1.28 points; *P* < 0.001) [18]. Long term extension of patients receiving treatment for 18 months demonstrated decreased progression on the NPC Clinical Severity Scale compared with expected natural-history decline, corresponding to about a 118% reduction in annual progression and sustained neurologic improvements, with good tolerability and no treatment-related serious adverse events [22]. However, limitations including study design, small sample size and durability make it uncertain to provide a definitive disease modifying conclusion. A phase 2 study showed that clinical improvements began to disappear after a 6-week washout period thus implying that benefit depends on continued therapy [21].

### GM2-Gangliosidoses

#### Epidemiology and Etiology

GM2-gangliosidoses are a group of rare autosomal recessive, genetically and clinically heterogeneous lysosomal storage disorders [6, 33]. With an approximate prevalence of 0.28 per 100,000 people, primarily affecting pediatric patients, it is prematurely fatal, especially in children experiencing the infantile form of GM2-gangliosidoses [19]. The most reported form is the infantile form with early-onset and presents from birth to around 10 months where patients have less than 5% residual enzyme activity leading to psychomotor regression, delay in developmental milestones, acoustic startle and irritability [34]. The juvenile form presents between the ages of 2–5 years, with progressive neurological deterioration and cerebellar ataxia following normal early development, while the late-onset form presents between the ages of 10–26 years with cerebellar ataxia, dystonia, and lower motor neuron disorder leading to proximal lower limb weakness [33, 35–37].

#### Pathophysiology

Gangliosides are complex glycolipids, abundant in brain tissue; approximately 5% of the brain’s gangliosides are GM2 gangliosides, generally found on the neuronal cell membrane. They are responsible for various functions including neuronal differentiation, myelin maintenance, cell signaling, membrane organization, inflammation, and cell adhesion [38–41]. The GM2 gangliosides are degraded by β-hexosaminidase A (*HexA*) and β-hexosaminidase B (*HexB*) in the lysosomes, along with the help of GM2 activator protein (GM2AP) that presents the GM2 ganglioside to HexA [34]. GM2-gangliosidoses results from mutation in either the *HEXA* gene responsible for α subunit of HexA and β-hexosaminidase S [HexS (αα)], or the *HEXB* gene responsible for β subunit of HexA and HexB (ββ), or the *GM2A* gene which codes for the GM2AP [38]. Mutation in these genes results in the inability of the lysosomes to degrade GM2 gangliosides, leading to gangliosidoses; Tay-Sachs disease (TSD) - the B variant (OMIM# 272800), Sandhoff disease (SD) - O variant (OMIM# 268800), or AB variant (OMIM# 272750) of GM2 gangliosidoses [34].

The accumulation of GM2 ganglioside in the neurons leads to several cytotoxic events, including demyelination, inflammation, and may even lead to neuronal death [33, 42, 43]. TSD patients have low HexA activity (mutation in the *HEXA* gene), SD patients have low HexA and HexB activity (mutation in the *HEXB* gene), while the AB variant is caused by a deficiency of GMP2AP [33, 44].

#### Clinical Manifestations

Patients with GM2 gangliosidoses have progressive neurological deterioration and motor deficits, hypotonia, vision impairment, delayed responsiveness, seizures, and progressive weakness, whereas patients with TSD may also present with hepatosplenomegaly, cardiomegaly, and skeletal abnormalities [35, 45, 46]. Approximately 200 mutations in the *HEXA* gene, 100 mutations in the *HEXB* gene, and around 10 mutations in the GM2A gene have been reported, including gross deletion, small deletion, splicing, missense and nonsense mutations. The heterogeneity in the onset and severity of the disease is determined by the residual enzyme activity [47–50].

#### Role of N-acetyl-L-Leucine in GM2-Gangliosidoses

A multinational, open-label, rater-blinded, phase IIb trial investigated the impact of NALL in 29 patients with GM2 gangliosidoses (TSD and SD), including children and adults. Patients were assessed before starting treatment with NALL, at the end of the 6-week treatment period and at the post-treatment washout visit; 6 weeks after stopping NALL. The primary endpoint of the study; clinical impression of change in severity (CI-CS) as measured by 9HPT or 8MWT, showed an improvement after the treatment, with a mean difference of 0.34 and after the washout period, the performance showed a decline with a mean decrease of −0.36. Ataxia was also assessed using the SARA protocol; the treatment showed a decrease of 1.41 points as compared to the baseline mean value of 14.24, indicating improvement in cerebellar function. Similar to CI-CS; the improvement in ataxia scores also reversed following the washout period with an increase of 1.43 points. The mDRS was used to measure overall neurological function and showed significant improvement but also worsened significantly following treatment discontinuation. Speech performance as measured by the PATA speech test showed improvement with the treatment and showed a significant decline during washout. Similar results were recorded for clinical global impression scores from the investigators, caregivers, and patients. Only 3 patients (10%) reported non-serious adverse effects, including asthenia, flatulence, tremor, and acne. These results established NALL as a promising oral therapeutic option for GM2 gangliosidoses [19].

#### Strengths and Weaknesses of N-acetyl-L-Leucine in Modifying Disease Course of GM2-Gangliosidoses

Observational and clinical research suggest potential disease modifying effect of NALL on GM2, with improvements in functioning and quality of life and a favorable safety profile without serious adverse reactions reported to date [51]. NALL demonstrated neuroprotective effects and improved gait in mouse models and selected human cases support the biological mechanism for modifying neurodegeneration [6]. However, GM2 gangliosidoses are ultra-rare, rapidly progressive disorders with no approved therapies, which restricts trial size and statistical power, making most available evidence derived from small cohorts, open-label designs, or observational data [19].

### Ataxia Telangiectasia

#### Epidemiology and Etiology

Ataxia telangiectasia (A-T), also known as Louis-Bar syndrome, is a rare autosomal recessive neurodegenerative disorder with an estimated prevalence of 1 in 40,000 to 100,000 live births worldwide [52–54]. The disorder usually appears in early childhood, with primary symptoms such as progressive cerebellar ataxia, oculomotor apraxia, and distinctive dilated blood vessels (telangiectasias), particularly in the skin and eyes. The phenotypic spectrum may extend beyond its classical presentation, given its substantial heterogeneity. Patients may present with absence of one or more of its classical features including ataxia, oculomotor apraxia or even telangiectasias [55].

#### Pathophysiology

The condition is caused by mutations in the A-T mutated (*ATM*) gene, which is found on chromosome 11q22-23, leading to neurological and systemic symptoms [56]. The *ATM* gene plays a central role in DNA damage response by facilitating the repair of DNA double-strand breaks (DSBs). It is activated by DSBs and coordinates repair mechanisms, cell cycle checkpoints, and apoptosis. ATM’s deficiency in A-T leads to impaired DNA repair, genomic instability, immune dysfunction, and neurodegeneration, contributing to the disease’s pathogenesis [57–60].

#### Clinical Manifestations

##### Neurological Features

A-T presents with the primary symptom of cerebellar ataxia, which progresses into significant neuromotor impairment because of cerebellar cortex degeneration. Patients also show telangiectasias, a main immunodeficiency, and different indications of early aging, such as thymus and gonadal degeneration, as well as growth delay [61]. Eye movement disorders occur in approximately 88% of A-T patients, appearing as saccadic dysmetria, difficulties with smooth pursuit, and issues with gaze-holding. These eye movement impairments are associated with cerebellar dysfunction and resulting abnormalities in the brainstem [62]. In addition to ocular motor issues, individuals with variant A-T frequently show movement disorders, such as dystonia, choreoathetosis, and tremors [63]. These movement disorders are often more evident than the cerebellar ataxia typical of classic A-T. Studies have shown that specific mutations in the *ATM* gene, especially missense mutations impacting the kinase domain, are linked to these serious movement disorders. This implies a genotype-phenotype relationship in which the remaining ATM kinase activity results in specific neuronal impairment, adding to the noted movement disorders [63].

##### Systemic Features and Risks

Associated with immunodeficiency, A-T accounts for increased susceptibility to cancer and early aging, with patients frequently suffering from a combination of neurological and systemic complications. These include motor and cognitive deficits, hormonal imbalances, and recurrent infections, leading to a substantial impact on QoL [55] and a high risk of lymphoid cancers and leukemias in younger patients and epithelial tumors in older adults. The illness is characterized by heightened sensitivity to ionizing radiation, rendering radiotherapy ineffective. Laboratory results show increased serum α-fetoprotein and carcinoembryonic antigen levels, diabetes with insulin resistance, and sporadic liver dysfunction [61]. These integrated characteristics emphasize the intricate nature of the illness, affecting both cellular reactions and localized tissue deterioration [64].

#### Atypical and Typical Presentation

In the article by Fortuna et al., a clinical case of a 12-year-old boy with A-T was presented, emphasizing the progressive characteristics of the illness. The patient showed developmental delay starting at 17 months, along with spasticity, ataxia, chorea, dyskinesia, and apraxia. At 12 years, telangiectasias were observed in the eye’s conjunctiva. Blood tests showed a deficiency of IgA and increased levels of alpha-fetoprotein. Molecular investigations verified the diagnosis by detecting two mutations in the *ATM* gene [65]. The research conducted by Teraoka et al. examined splicing abnormalities in the *ATM* gene, showing that about 48% of mutations in individuals with A-T impacted RNA splicing. Although certain mutations changed the typical GT (donor) or AG (acceptor) splice sites, numerous others affected less conserved sequences, resulting in exon skipping, retention of introns, or activation of hidden splice sites. In numerous instances, nonsense and missense mutations were linked to exon skipping, highlighting the intricacy of ATM splicing regulation. Western blot analysis revealed that the ATM protein was absent in all the mutant cell lines, indicating that these splicing defects resulted in a total loss of protein function. Notably, several exon-skipping occurrences were observed in normal controls as well, emphasizing the necessity for careful interpretation of cDNA-based mutation analyses lacking genomic validation. This research highlights the important impact of splicing mutations in AT and the need for a thorough genomic evaluation for a precise diagnosis [66].

#### Roleof N*-*acetyl*-*L*-*Leucine in Ataxia Telangiectasia

Given the progressive nature of A-T, therapeutic interventions primarily focus on managing symptoms and slowing down the degenerative process. One promising therapeutic agent under investigation is NALL. NALL has shown some potential in stabilizing motor functions in individuals with cerebellar ataxia, including those with A-T.

The clinical study by Beyraghi-Tousi et al. included only a very small cohort of patients with A-T, with no formal power calculation, making it a significantly underpowered case series. The compound used was not Good Manufacturing Practice (GMP) quality but obtained from an online generic Chinese manufacturer. The six-week treatment period (NALL 3 g/day) was tolerated well and demonstrated improvements in gastrointestinal symptoms, including constipation and nausea. Some secondary benefits, such as enhanced mobility for daily activities, were also observed, but no significant gains were noted in the main clinical ataxia measures such as the SARA scale, 9HPT, or the 8MWT. Given the limitations of the study conduct (non-GMP or Good Clinical Practice [GCP]), the results of the trial have limited interpretability. Although the initial results were not definitive, one suggestion is that longer treatment durations (16 weeks or more) could have clinical advantages. This suggests that NALL could exert benefits over longer periods by facilitating neuroprotective processes, stabilizing oxidative stress factors, and improving mitochondrial activity in neurons. Considering that A-T is marked by considerable oxidative stress and mitochondrial dysfunction, these characteristics of NALL might be advantageous in reducing neuronal damage over time [50].

#### Strengths and Weaknesses of N-acetyl-L-Leucine in Modifying Disease Course of Ataxia Telangiectasia

Preliminary data suggest mixed effects of NALL in improving the disease course. In a recent study, NALL did not significantly improve primary motor outcomes however, it did benefit the non-motor effects, indicating good tolerability but limited proof of direct neurodegeneration-slowing efficacy [23]. A case study of N-acetyl-DL-leucine in a child with A-T showed progressive improvement in Scale for the Assessment and Rating of Ataxia (SARA) scores and quality-of-life measures over 16 weeks, suggesting symptomatic and possibly disease-modifying benefit, though conclusions are limited by single-patient design [67].

### Multiple sclerosis

#### Epidemiology and Etiology

MS is a complex, unpredictable neurological condition characterized by chronic inflammation, immune system dysregulation, and degenerative changes [68]. MS is globally prevalent, affecting approximately 2.3 million people from North America and Europe, with the highest prevalence, to Asian and Sub-Saharan African countries having the least prevalence [64]. The global incidence of MS is probably increasing irrespective of the regional prevalence [69]. MS targets young adults ranging between 20 years and 40 years of age. Women are 2 to 3 times more susceptible to developing MS than males due to hormonal imbalance, suggesting its hormonal predisposition. Other factors that predispose to this condition include the environment (vitamin D deficiency), genetics (associated with the HLA-DRB1*15:01 allele), Epstein-Barr Virus and cigarette smoking [70–72].

#### Pathophysiology

MS occurs due to the infiltration of auto-reactive B-cells, T-cells, and macrophages in the CNS [73, 74]. These changes lead to the damage of the protective myelin sheath (demyelination) and the nerve fibers themselves (axonal damage), resulting in the disruption of normal neural transmission, formation of glial scar by astrocytes, and focal demyelinating plaques, which can be observed on the periventricular white matter, brainstem, and spinal cord on magnetic resonance imaging (MRI) [68, 72, 75]. Crucial molecular elements in the pathophysiology of MS are myelin basic protein (MBP), interleukin-17 (IL-17), and tumor necrosis factor-alpha (TNF-α) which causes impairments of physical, cognitive, and psychological abilities and a range of debilitating symptoms [68].

#### Clinical Manifestations

The clinical manifestations of MS include a wide range of symptoms, including motor disturbances (muscle weakness, spasticity and neurogenic gait), sensory disturbances (tingling sensation and numbness), visual disturbances (diplopia and optic neuritis), autonomic disturbances (inability to control bladder and bowel functions) as well as cognitive and psychiatric disturbances (anxiety, depression and impairments in memory processing and speed) [69, 71]. There is also a specific relapse-remission pattern of symptoms that showcases Lhermitte’s sign (electric-shock sensation with neck flexion) and Uhoff’s phenomenon (symptom worsening with heat) [69]. Other than MRI, further diagnosis can be processed by examining the cerebrospinal fluid through lumbar puncture, which displays oligoclonal immunoglobulin IgG bands found in up to 90% of patients suffering from MS [75].

#### Role of N-acetyl-L-Leucine in Multiple Sclerosis

Management of multiple sclerosis (MS) generally involves relapse therapy, disease-modifying treatments, and symptomatic medications [76]. Although NALL has not yet been tested in MS clinical trials, experimental and limited clinical data suggest potential neuroprotective mechanisms.

In the experimental autoimmune encephalomyelitis model, NALL reduced limb paralysis and preserved myelin integrity [72]. It has been shown to attenuate oxidative stress and modulate excitatory neurotransmission, supporting neuronal energy metabolism and limiting reactive oxygen species-mediated injury [77]. NALL also promoted vestibular compensation and postural control in animal studies [78]. In human induced primary neurons derived from individuals with MS, NALL upregulated glutamate-cysteine ligase (GCL), NRF2, and NQO1, enhancing glutathione-mediated antioxidant defense and maintaining neuronal structure under stress [79].

Clinically, a single report described an MS patient with acquired pendular nystagmus who experienced improved visual stability with NALL and memantine [80]. A phase II trial in cerebellar ataxias similarly demonstrated improved coordination and reduced fatigue [81]. Although derived from other neurological contexts, these findings highlight mechanisms that may be relevant to MS, warranting further controlled studies.

#### Strengths and Weaknesses of N-acetyl-L-Leucine in Modifying Disease Course of Multiple Sclerosis

Evidence supporting strength of disease modifying effect of NALL in MS is extremely limited and mostly relates to the symptomatic effects. A case report showed improvement in ocular motor symptoms, suggesting possible functional neurologic benefit but not demonstrating any change in disease progression or inflammatory activity in patients with MS-related acquired pendular nystagmus [80]. The main theoretical strength supporting NALL as a disease-modifying candidate in MS is its demonstrated neuroprotective and anti-injury effects in preclinical neurologic models, such as improved recovery and reduced neuronal damage after brain injury [12].

### Parkinson’s Disease

#### Epidemiology and Etiology

PD is the second most common degenerative neurological disorder with gradual progression. Individuals under 40 years of age are less susceptible to this disease. The global prevalence of PD is approximately 7 to 10 million, with men being more prone to this disease than women [82]. With a steep incline in its incidence worldwide from 1995 to 2015, the numbers are projected to double again by 2030, highlighting the urgency to find preventive and curative measures [83].

Although PD is believed to be idiopathic, some factors of this disease are environmental and genetic. Genetic mutations are found in only 5–10% of cases, indicating that they are mostly affected by environmental and behavioral aspects. However, stress and aging are major agents in the development of PD [84].

#### Pathophysiology

Pathology occurs in Substantia nigra pars compacta where the disintegration of dopaminergic neurons is observed, leading to a deficiency of dopamine in the striatum. Lewy bodies are pathognomonic for PD and are formed by the accumulation of alpha-synuclein proteins that are misfolded [82, 85]. Systems responsible for regulating disintegrated proteins such as ubiquitin-proteasome are also affected. Neuroinflammation has also been observed, which facilitates neurodegeneration in PD [86]. According to multiple studies, several mutations may lead to early-onset autosomal recessive PD which include the *PARKIN* and *PINK1* genes (abnormal mitochondrial function and ROS), *DJ 1* gene (normally has antioxidant effects) and the *LRRK2* gene (elevates kinase enzyme activity) [87–90].

#### Clinical Manifestations

Clinically, PD is exhibited by the disruption in both motor control and autonomic functions. Disruption in motor control includes bradykinesia (slow movements), postural imbalance, muscle stiffness, and resting tremors. Disruption in autonomic functions includes cognitive disability (memory loss), depression, anxiety, insomnia or sleep disorder, and constipation. PD in a patient can be assessed by trial therapy of levodopa or MRI [91]. Decreased PGC-1 alpha levels also indicate PD, if not taken care of at the early stages, may progress to highly complicated medical manifestations such as dyskinesia, hallucinations and memory loss [92].

#### Role of NALL in Parkinson’s Disease

PD is a chronic condition without a cure, but treatment options are available to mitigate its symptoms which include levodopa, dopamine agonists, monoamine oxidase inhibitors, and NALL being one of them. In a case report published in 2024, 2 individuals suffering from REM sleep behavior disorder (also seen in PD) were treated with NALL. Over 22 months, it was observed that disease advancements were arrested and showed positive changes in key disease indicators, suggesting that NALL is a promising agent in preventing disease progression in the early stages [93]. Researchers suggest that NALL helps control neuronal excitability and safeguards neurons by reinforcing cellular membranes and limiting excitotoxicity; factors that underline its advantages in neurodegenerative disorders [12].

PD, being recognized within highly heterogeneous clinicopathological spectrum, shows distinct motor phenotypes, including tremor-dominant and akinetic-rigid subtypes, non-motor burden, difference in progression rate and therapeutic response. These factors challenge the uniform treatment paradigm and support the precision medicine model. In this regard, the role of NALL should be interpreted within the context of disease heterogeneity, with the possibility that its benefits may be confined to certain subtypes [94].

A recent preclinical study by Xu et al. showed that NALL significantly improves PD-like symptoms in MPTP-treated mouse models by suppressing Desulfobacterota, a group of bacteria associated with inflammation in the gut microbiome. By modulating the gut-brain axis, NALL reduces neuroinflammation and neuronal degeneration, presenting itself as a promising approach for improving both the motor and non-motor symptoms of PD [20]. While the specific role of NALL in PD needs to be studied and researched further, several studies indicate improved dopaminergic functions in PD patients by N-Acetyl compounds by increasing dopamine transport, further suggesting NALL to be a future candidate for symptom-managing agent in PD [95].

#### Strengths and Weaknesses of N-acetyl-L-Leucine in Modifying Disease Course of Parkinson’s Disease

Pre-clinical studies demonstrate possible disease modifying potential of NALL in Parkinsonian neurodegeneration. In experimental models, NALL improves synaptic function and upregulates lysosomal, mitochondrial, and synaptic proteins in patient-derived dopaminergic neurons, mechanisms consistent with neurorestorative and disease-slowing activity [9]. However, there is a predominance of animal studies and only selective human cases thus causing a reliance on surrogate biomarkers rather than progression endpoints.

### Multiple Sulfatase Deficiency

#### Epidemiology and Etiology

Multiple sulfatase deficiency (MSD), also known as Austin disease, is a rare lysosomal storage disorder with an estimated prevalence ranging from 1 in 100,000 to 1 in 500,000 births [94]. According to recent reports, only around 150 cases have been documented worldwide [94]. The underlying cause of MSD is an autosomal recessive mutation in the *sulfatase-modifying factor 1* gene, located on chromosome 3p26.1, which encodes the formylglycine-generating enzyme (FGE) [96, 97].

#### Pathophysiology

FGE is required for the modification of all sulfatases after translation [97]. Sulfatases are a group of enzymes involved in the breakdown of molecules containing sulfates, such as sulfolipids, glycosaminoglycans, and steroid sulfates [98]. The mutation alters the function of FGE, and the lack of functional FGE in MSD leads to the impaired activity of all sulfatases, resulting in the lysosomal accumulation of sulfated substrates in various tissues and organs, causing widespread cellular dysfunction [97].

#### Clinical Manifestations

MSD presents a spectrum ranging from severe neonatal onset to milder juvenile forms. The neonatal form is characterized by rapid onset with severe systemic and neurological dysfunction, and life expectancy is often limited to the first 2 years [97]. The late infantile form is the most common subtype of MSD, where initial normal development is followed by progressive regression, whereas the juvenile form is the rarest variant, marked by delayed symptom onset and a subacute disease course [97]. The common clinical manifestations of MSD include developmental regression, skeletal deformities, organomegaly, hearing loss, cardiac valve disease, and neurological deterioration [22, 97].

#### Role of N-acetyl-L-Leucine in Multiple Sulfatase Deficiency

There is no available disease-modifying therapy for MSD, however, a crossover case study has explored the effect of NALL on the improvement of symptoms in a patient with MSD based on the anti-inflammatory action of NALL in the cerebellum [22]. A 12-year-old girl with MSD, confirmed through genetic testing, received a 3 g/day dose of NALL. There was major improvement in the ataxia symptoms with a 25% reduction in the SARA score from 20 to 15 after treatment with NALL [22]. The gait significantly improved and upon evaluation with SCAFI score, 8MWT decreased from 8.71 to 7.93 s [22]. The QoL index increased from 74.21 to 82.65 along with an improvement in social and emotional functioning [22]. Additionally, the energy intake also showed an increment throughout the period of treatment. There were no concerning side effects and NALL was tolerated well by the patient, further confirming its safety and efficacy [22].

This study highlights the role of NALL as a symptomatic treatment option in patients with MSD; however, further research and studies are necessary to validate these findings and establish the full therapeutic applicability of NALL in patients with MSD.

#### Strengths and Weaknesses of N-acetyl-L-Leucine in Modifying Disease Course of Multiple Sulfatase Deficiency

A single clinical crossover case study has demonstrated that NALL improved neurological function and quality-of-life measures in a patient with this progressive neurodegenerative lysosomal disorder, which is characterized by worsening psychomotor decline and neurologic deterioration [24]. Available data derived from single-patient studies without controls, objective progression biomarkers, or long-term follow-up, makes it impossible to conclude whether NALL truly alters the natural history of MSD.

## From Acetyl-DL-Leucine to N-acetyl-L-Leucine: Lessons from Early Trials

While NALL has gathered increasing attention for its promising therapeutic potential in a variety of neurological disorders, it is important to acknowledge the earlier use of its racemic predecessor acetyl-DL-leucine and the limitations observed in clinical studies. Acetyl-DL-leucine is a 1:1 mixture of two enantiomers, D-leucine and L-leucine, but only the L-isomer is biologically active [6]. Initially approved for the symptomatic treatment of vertigo, acetyl-DL-leucine was subsequently explored in neurodegenerative conditions, yet with limited success.

A case series by Scigliuolo et al. evaluated the effects of acetyl-DL-leucine in patients with multiple system atrophy of the cerebellar type (MSA-C), a rare neurodegenerative disorder of late onset marked by autonomic dysfunction and prominent cerebellar impairment. There were no clinically meaningful improvements in motor symptoms assessed through the Timed Up and Go test and 9HPT [99]. Similarly, the ALCAT trial, assessing the efficacy of acetyl-DL-leucine in cerebellar ataxias, also failed to demonstrate statistically significant improvements in key outcome measures such as the SARA and SCAFI z scores [100].

These negative findings underscore the limitations of the racemic compound and highlight the importance of stereospecificity in drug development. The lack of efficacy observed with acetyl-DL-leucine likely reflects both the pharmacologically inactive nature of the D-enantiomer and its potential to interfere with the desired biological effects of the L-isomer [78]. These unsuccessful trials provided a pivotal rationale for the development and investigation of the enantiomerically pure NALL, which has since shown more favorable pharmacokinetics, better blood-brain barrier penetration, and positive clinical efficacy in randomized controlled trials for inherited and acquired neurological disorders.

## Safety Profile of N-acetyl-L-Leucine

The established safety profile of NALL is favorable, with minimal adverse effects reported, and its good tolerability supports long-term use and higher dosing protocols. NALL is strongly supported by clinical trials in providing symptomatic treatment for NPC as it provides significant and clinically meaningful improvement in the QoL and neurological function and is regarded as safe and tolerable. In these patients, NALL demonstrated mild treatment-emergent adverse effects including diarrhea, flatulence, increased hunger, aggressive behavior, and rash but the incidence of such events usually remained low; under 10% of the participants [21, 80].

Similarly, clinical studies affirm the safety and tolerability of NALL at doses up to 4 g/d in GM2-gangliosidoses patients. Only flatulence, acne, tremors, and asthenia were reported in 10% of the patients without any serious adverse events, thus proving further evidence in the general acceptability of the drug in such patients [19, 51]. Furthermore, NALL has shown promise as a potential treatment for AT, with reported improvements in ataxia, ocular stability, and QoL and no life-threatening adverse effects [101]. Its efficacy, safety, and ability to target key neurological deficits position NALL as a viable therapeutic option for progressive neurodegenerative disorders with limited treatment options [19, 52, 101].

Clinical efficacy of NALL in the treatment of PD, MSD and MS underscores the need for further trials to potentiate the safety profile of the drug with these individuals, however, present studies demonstrate no treatment-emergent adverse effects but symptomatic improvements in neurological function and reduced morbidity [20, 22]. Moreover, NALL is also a P-glycoprotein (P-gp) inhibitor and can affect elimination of substances dependent on P-gp efflux thus caution is advised when using P-gp substrate drugs including digoxin and dabigatran, etc [102]. NALL provides a favorable benefit risk profile for the treatment of neurological disorders, and with no approved therapeutically safe and tolerable treatment choice available, it presents as a promise for these debilitating diseases.

## Conclusion

In conclusion, NALL has emerged as one of the most promising treatment options for neurodegenerative and neuroinflammatory conditions, especially due to its unique poly-pharmacological mechanism of action that addresses hallmark cellular processes of neurodegeneration that are shared across a broad range of neurological disorders. Its advantageous pharmacokinetics, improved blood-brain barrier penetration, and stereospecific biological effects have played a role in its noted clinical advantages, especially in lysosomal storage disorders such as NPC and GM2 gangliosidoses. Despite the drawbacks linked to its racemic counterpart, acetyl-DL-leucine, the shift to the enantiomerically pure L-form highlights the significance and importance of accuracy in drug development. Initial and current clinical trials indicate that NALL provides both symptom relief and possible disease-modifying benefits, yet comprehensive long-term data are still required. Ongoing investigation into NALL’s modes of action, ideal treatment lengths, and therapeutic combinations will be essential in clarifying its position in contemporary neurology and broadening its use to other presently incurable neurological disorders.

## Data Availability

No datasets were generated or analysed during the current study.
